# Lord Rosebery on Hospitals

**Published:** 1899-09-16

**Authors:** 


### LORD ROSEBERY ON HOSPITALS.

When speaking at Bishop Auckland last week, on the occasion of opening a cottage hospital, Lord Rosebery eulogised the advantages of hospital treatment in the following short sentence: "Everybody, from the king to the beggar, is in illness better treated in a hospital than in his own home, and the reason is very simple, because the home is meant for health and the hospital for illness." On this little sentence it would not be difficult to preach a sermon which might perhaps be of some use to certain would-be hospital reformers, who, when attempting to deal with what is so often spoken of as hospital abuse, fail to see that the crowds of more or less well-to-do sick who press forward to obtain the benefits of hospital treatment do so not altogether, as these reformers would have us believe, from a mean-spirited desire to get their medical attendance on the cheap, but from a conviction that at the hospitals they will obtain what they cannot obtain elsewhere. In every community there are, of course, plenty of cheeseparing and stingy people who begrudge every payment, and who will endeavour by hook or by crook to get hold of whatever is to be had for nothing; and we go heartily with those who, by careful investigation of the circumstances of hospital patients, by inquiry officers, and by other machinery, would turn adrift such parasites of our hospital system. But when it is maintained, as we hear it constantly maintained, that a man who "can afford to pay a doctor" is from that fact an unfit case for hospital treatment, we know that the criterion is wrong, and that the ability to pay a doctor has but little to do with the question at issue. Lord Rosebery's remark gives the key to the situation. It is the widespread belief in the superiority of the help given by the hospitals which is the cause of their attractiveness to that very class whose presence among hospital patients most excites the ire of hospital reformers. Probably these reformers would make more way if, instead of concentrating their energies on keeping out those who, according to an arbitrary standard, are unfit, they would invent some system by which deserving cases, even although able to pay a little, might be admitted to our hospitals on some self-respecting basis.

